# Supplementary material for: The influence of biological, epidemiological, and treatment factors on the establishment and spread of drug-resistant Plasmodium falciparum
Source: eLife. 2022 Jul 7;11:e77634. doi: 10.7554/eLife.77634 (PMC9262398; doi:10.7554/eLife.77634)
Supplement: Transparent reporting form [file elife-77634-transrepform1.docx]

***eLife’s* transparent reporting form**

We encourage authors to provide detailed information *within their submission* to facilitate the interpretation and replication of experiments. Authors can upload supporting documentation to indicate the use of appropriate reporting guidelines for health-related research (see [EQUATOR Network](http://www.equator-network.org/%20)), life science research (see the [BioSharing Information Resource](https://biosharing.org/)), or the [ARRIVE guidelines](http://www.plosbiology.org/article/info:doi/10.1371/journal.pbio.1000412) for reporting work involving animal research. Where applicable, authors should refer to any relevant reporting standards documents in this form.

If you have any questions, please consult our Journal Policies and/or contact us: [editorial@elifesciences.org](mailto:editorial@elifesciences.org).

**Sample-size estimation**

- You should state whether an appropriate sample size was computed when the study was being designed
- You should state the statistical method of sample size computation and any required assumptions
- If no explicit power analysis was used, you should describe how you decided what sample (replicate) size (number) to use

Please outline where this information can be found within the submission (e.g., sections or figure legends), or explain why this information doesn’t apply to your submission:

This section does not apply to our study because we did not collect data. In this study, we have developed a disease modelling approach with emulator-based global sensitivity analysis to systematically quantify which factors drive the establishment and spread of drug-resistant Plasmodium falciparum parasites.

We simulated the spread of drug-resistant parasites in a simulated population composed of 100’000 humans. We used a large population size to minimize the stochastic fluctuation of allele frequency, also called genetic drift (as shown by Hasting et al. (2020)). Reducing the strength of genetic drift made the analysis more efficient by minimizing the risk that the resistant genotype becomes extinct due to stochastic extinction and by increasing the accuracy of the estimated rate of spread (see Methods: simulate and estimate the rate of spread of the drug-resistant genotype).

We used a simulated human population size of 10’000 humans when we assessed the probability of establishment of resistant parasites. In this case, we used a smaller population size than our previous simulations as we did not aim to minimise the influence of genetic drift but to assess its effect on the establishment of drug-resistant parasites. Using a smaller population size made the process more efficient (see Methods: establishment of drug resistance).

**Replicates**

- You should report how often each experiment was performed
- You should include a definition of biological versus technical replication
- The data obtained should be provided and sufficient information should be provided to indicate the number of independent biological and/or technical replicates
- If you encountered any outliers, you should describe how these were handled
- Criteria for exclusion/inclusion of data should be clearly stated
- High-throughput sequence data should be uploaded before submission, with a private link for reviewers provided (these are available from both GEO and ArrayExpress)

Please outline where this information can be found within the submission (e.g., sections or figure legends), or explain why this information doesn’t apply to your submission:

Biological and technical replicates do not apply to our study as we did not collect data.

For our model-based analysis –

1) When we assessed the rate of spread, each simulation was performed in five stochastic realizations to assess the impact of model stochasticity on the output (see Methods: simulate and estimate the rate of spread of the drug-resistant genotype).

2) When we assessed the probability of establishment of a drug-resistant mutation, each simulation was performed in 300 stochastic realizations due to the high stochasticity of this step (see Methods: establishment of drug resistance).

3) We included all simulation data generated by the model for each analysis.

We did not observe outliers and did not use High-throughput sequence data.

**Statistical reporting**

- Statistical analysis methods should be described and justified
- Raw data should be presented in figures whenever informative to do so (typically when N per group is less than 10)
- For each experiment, you should identify the statistical tests used, exact values of N, definitions of center, methods of multiple test correction, and dispersion and precision measures (e.g., mean, median, SD, SEM, confidence intervals; and, for the major substantive results, a measure of effect size (e.g., Pearson's r, Cohen's d)
- Report exact p-values wherever possible alongside the summary statistics and 95% confidence intervals. These should be reported for all key questions and not only when the p-value is less than 0.05.

Please outline where this information can be found within the submission (e.g., sections or figure legends), or explain why this information doesn’t apply to your submission:

We developed an emulator- and disease model-based approach to systematically quantify the influence of multiple factors on the establishment and spread of drug-resistant parasites. In essence we undertook a global sensitivity analysis with an emulator of our computational expensive individual based model of malaria. The steps of our analysis are detailed in the Methods of our paper.

In summary, we performed each global sensitivity analysis on an emulator trained on a large number of model simulations. We assessed the fit of the emulator as follows: For each global sensitivity analysis, we simulated a range of between 700 to 2300 parameter combinations with five stochastic realizations (thus a total of 3500 to 11,500 simulations for each analysis – and the number varied depending on the number of simulations needed to reach a satisfactory fit of the emulator). The emulator was fitted using a training dataset containing 80% of simulations. The remaining simulations (20%) were kept performing an out-of-sample comparison analysis. In this analysis, we assessed the accuracy of the emulator by estimating the correlation coefficient, and the root means squared error between the selection coefficients predicted with the emulator and the expected selection coefficients (estimated with our model) (see Methods: train the emulator and improve its accuracy).

(For large datasets, or papers with a very large number of statistical tests, you may upload a single table file with tests, Ns, etc., with reference to sections in the manuscript.)

**Group allocation**

- Indicate how samples were allocated into experimental groups (in the case of clinical studies, please specify allocation to treatment method); if randomization was used, please also state if restricted randomization was applied
- Indicate if masking was used during group allocation, data collection and/or data analysis

Please outline where this information can be found within the submission (e.g., sections or figure legends), or explain why this information doesn’t apply to your submission:

This section does not apply to our study, as we did not collect data nor allocate samples into experimental groups.

**Additional data files (“source data”)**

- We encourage you to upload relevant additional data files, such as numerical data that are represented as a graph in a figure, or as a summary table
- Where provided, these should be in the most useful format, and they can be uploaded as “Source data” files linked to a main figure or table
- Include model definition files including the full list of parameters used
- Include code used for data analysis (e.g., R, MatLab)
- Avoid stating that data files are “available upon request”

Please indicate the figures or tables for which source data files have been provided:

All data and code used to produce the figures are available at https://zenodo.org/badge/latestdoi/458226427.

In addition, the code used to run the simulations and perform the analyses can be found at https://zenodo.org/badge/latestdoi/458217287.

The individual-based model of malaria transmission and epidemiology used in the study has an open-access code (https://github.com/SwissTPH/openmalaria) and documentation (https://github.com/SwissTPH/openmalaria/wiki).

Note that we did not provide the data and codes used to produce Figure 1, Figure 3–figure supplement 6, Supplementary file 1**–**figure 2, Supplementary file 1–figure 4, and Supplementary file 1**–**figure 13 because these figures were illustrations and not plots of simulation results.
